# Characterizing Microbial Signatures on Sculptures and Paintings of Similar Provenance

**DOI:** 10.1007/s00248-020-01504-x

**Published:** 2020-05-21

**Authors:** Manolito G. Torralba, Claire Kuelbs, Kelvin Jens Moncera, Rhonda Roby, Karen E. Nelson

**Affiliations:** 1 J. Craig Venter Institute, 4120 Capricorn Lane, La Jolla, CA 92037 USA; 2 Alameda County Sheriff’s Office, Crime Laboratory, 2901 Peralta Oaks Court, Oakland, CA 94605 USA

**Keywords:** Genomics, Sequencing, Microbiome, Microbial ecology, Artwork, Environmental microbiology

## Abstract

The preservation of artwork challenges museums, collectors, and art enthusiasts. Currently, reducing moisture, adjusting the type of lighting, and preventing the formation of mold are primary methods to preserving and preventing deterioration. Other methods such as ones based in detailed knowledge of molecular biology such as microbial community characterization using polymerase chain reaction (PCR) and sequencing have yet to be explored. Such molecular biology approaches are essential to explore as some environmental bacteria are capable of oxidizing nonpolar chemical substances rich in hydrocarbons such as oil-based paints. Using 16S rDNA Illumina Sequencing, we demonstrate a novel finding that there are differing bacterial communities for artwork from roughly the same era when comparing paintings on wood, paintings on canvases, and sculptures made of stone and marble. We also demonstrate that there are specific genera such as *Aeromonas* known for having oxidase positive strains, present on paintings on wood and paintings on canvas that could potentially be responsible for deterioration and fading as such organisms produce water or hydrogen peroxide as a byproduct of cytochrome c oxidase activity. The advantages of these genomics-based approaches to characterizing the microbial population on deteriorating artwork provides immense potential by identifying potentially damaging species that may not be detected using conventional methods in addition to addressing challenges to identification, restoration, and preservation efforts.

## Introduction

Works of art, from the Renaissance period, for example have had an important influence in nearly all aspects of human creativity, innovation, and imagination. From inspiring architecture and design to influencing human interactions, artwork has become an integral part of society. From a financial perspective, art sales have grown into a multibillion dollar industry with total worldwide sales of over $63.8 billion dollars in 2015, 43% of which were in the USA alone [1]. The profitability of such an industry comes with its challenges, particularly with the restoration and preservation of damaged and/or aging artworks where restoration efforts for various sized individual paintings can range from US $1000 to $15,000 [2]. Some of the necessary equipment used for these efforts can multiply these costs, such as sophisticated x-ray machines and infrared cameras costing $100,000 each [2]. Additionally, authenticity in this industry is becoming more and more challenging as counterfeits today are more sophisticated [3, 4]. Restoration and preservation efforts are increasingly important to museums and art collectors as the value of artwork continues to climb ([5, 1]).

Currently, most restoration and preservation efforts address physical and chemical aspects with minor emphasis on the effect of microorganisms when it comes to preservation ([5]). Limiting excess exposure to direct sunlight, heat sources, UV light, and moisture are all highly recommended [6]. The most common preservation and restoration effort regarding microorganisms is the preventing the formation of fungal colonies commonly observed as mold. Preventive measures against mold utilized by museums include monitoring humidity and reducing exposure to natural light and moisture [6, 7]. Efforts to characterize microbial communities using thorough approaches such as genomics on artwork have yet to be thoroughly conducted.

Initial studies using molecular biology techniques such as DGGE, Sanger sequencing, and culture-dependent approaches have shown that microorganisms belonging to three phyla, *Actinobacteria*, *Proteobacteria*, and *Clostridia*, are detected in deteriorating artwork and may be implicated in the rapid decay of fresco, concrete, marble, sandstone, and murals [8, 9, 10, 11, 12, 13]. Paintings provide a unique substrate for microbes to grow as paints often contain a variety of biodegradable organic and inorganic compounds that can be exploited by microorganisms as a source of nutrients [14, 15, 10, 16, 17]. Additionally, the substrate material that is often used for paintings can also provide biodegradable material such as animal or plant glues used for support, and cellulose in paper, canvas, and wood [14]. It is clear that these types of studies are gaining importance as we now know that specific bacteria are known to degrade environmental hydrocarbons commonly found in oil-based paints as well as produce various acids as metabolic end products [18, 19]. Though it is tempting to speculate that these bacterial processes are likely associated with the rapid decay of artwork, it is premature since studies focusing on entire bacterial communities and potential associated metabolic processes using next generation sequencing have yet to be conducted prior to our study.

In order to fully characterize the effect that microbial communities have on degradation of aging artwork, henceforth referred to as biodegradation, comprehensive genomics approaches are suggested as sequencing costs have dramatically reduced and large datasets on this sample type will provide a novel perspective on identifying entire communities. With the use of Illumina sequencing and conserved genetic markers such as 16S rRNA, we have been able to characterize entire microbial communities on various specimens of aging artwork. Our study characterizes the microbial communities on stone/marble, wood, and canvas from a private collection near Florence in the Tuscany region of Italy. Though prior studies have attempted to characterize the microbial composition associated with artwork decay, our results summarize the first large scale genomics-based study to understand the microbial communities associated with aging artwork.

## Experimental Procedures

### Sampling of Artwork

**Fig. 1:**
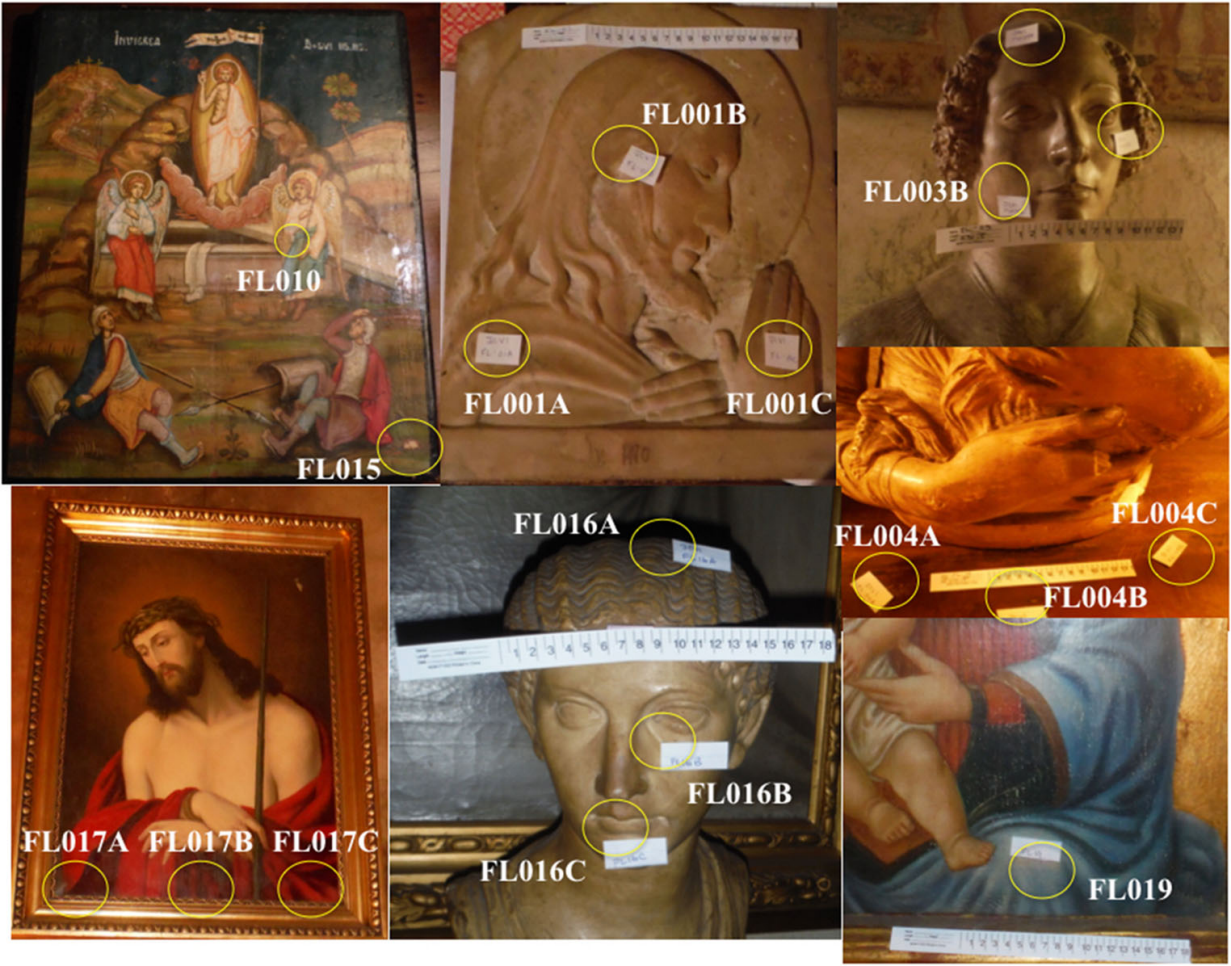
Photo summary of the various artworks sampled for this study. Circles indicate swabbed areas on each sample artwork. Photos not shown include: FL007B and FL002B

Several pieces from a private art collection in the Tuscany region of Italy were used as samples for our study (Fig. 1 and Table 1). The private collection was stored in an approximate 2000 sq.ft. area with low lighting and an ambient cool environment. While we have the assurance of the owner of the private collection that the works have been handled little in recent decades and never restored, we did not obtain documentation of the provenance of the works. Five of the six works are in a Renaissance style, although it is possible that they may have been created later. One piece is in a Roman style but may also be from the Renaissance or later. Recent history of ownership suggests the works are at least a couple of centuries old, if not much older. For our proof-of-concept study, the key was to have access to a variety of older artwork substrates that had not been aggressively cleaned.

**Table 1:** Summary of samples collected and processed. All samples with the exception of FL019 were stored in the same room

Sample ID	Data collected	Collection location	Substrate	Comment/description
FL001A	11/30/15	Tuscany, Italy	Marble/stone	Pink marble sculpture-deep swab of shoulder area
FL001B	11/30/15	Tuscany, Italy	Marble/stone	Pink marble sculpture-deep swab of temple and ear
FL001C	11/30/15	Tuscany, Italy	Marble/stone	Pink marble sculpture-deep swab of hand
FL002B	11/30/15	Tuscany, Italy	Marble/stone	Deep swab of shelf floor
FL003B	11/30/15	Tuscany, Italy	Marble/stone	Renaissance-style bust-dining room-deep swab of left cheek
FL016A	12/1/15	Tuscany, Italy	Marble/stone	Roman-style bust-deep swab of hair
FL004A	11/30/15	Tuscany, Italy	Wood	Deep swab of wood cabinet
FL004B	11/30/15	Tuscany, Italy	Wood	Deep swab of wood cabinet
FL004B	11/30/15	Tuscany, Italy	Wood	Deep swab of wood cabinet
FL007B	12/1/15	University of Firenze	Wood	Ascension-light swabbing of black paint-painted side
FL010	12/1/15	University of Firenze	Wood	Ascension-deep swirl
FL015	12/1/15	Tuscany, Italy	Wood	Ascension-deep swab of large broken patch of exposed wood
FL017A	12/1/15	Tuscany, Italy	Canvas	Christ painting-deep swab bottom left
FL017B	12/1/15	Tuscany, Italy	Canvas	Christ painting-deep swab bottom center
FL017C	12/1/15	Tuscany, Italy	Canvas	Christ painting-deep swab bottom right
FL019	12/1/15	Tuscany, Italy	Canvas	Madonna painting deep swab over green spots possible biofilm formation

The sampled artwork varied in substrates that ranged from paintings on wood, paintings on canvas, and stone and marble sculptures. The samples were collected using sterile techniques in preparation for DNA extraction, 16S PCR and Illumina Sequencing. Individually wrapped sterile swabs (Ref 25-806-1PD, Puritan Medical Products, Guilford, ME) were used to swab various sections on each art piece with approximately 10–12 gentle swipes per collection of an approximate 3-cm^2^ area. The sample swab tips were transferred to 1.5-mL sterile microcentrifuge tubes. A total of 500 μL 1X TE buffer was also added to each tube containing a swab tip. Each art piece was sampled in three distinct areas with at least 3 cm between each sampled section. Negative controls were collected by opening swab packages in the same location as the collected swab samples and immediately placing swabs into collection tubes with 1X TE buffer. A summary of the samples is outlined in Table 1 and shown in Fig. 1.

### DNA Extraction, 16S PCR, and Sequencing

Each sample swab was treated with 700 μL lysis buffer (20 mM Tris-Cl, pH 8.0, 2 mM EDTA, 1.2% Triton X-100) and incubated at 75 °C for 10 min. Samples were then cooled to room temperature, treated with 200 mg/mL lysozyme (Sigma/Aldrich, St Louis, MO) and incubated at 37 °C for 60 min, followed by adding 100 μL 10% SDS and 20 mg/mL proteinase K (Life Technologies, Carlsbad, CA) and incubated at 55 °C overnight. DNA was twice extracted from the lysate by an equal volume of phenol chloroform isoamyl alcohol followed by ethanol precipitation. Extracted DNA was suspended in 1X TE buffer. Residual PCR inhibitors were removed using the MOBio Powerclean kit (MOBio Labs, Carlsbad, CA) using manufacturer’s specifications. DNA was quantified using fluorometric methods (SybrGold, ThermoFisher, Waltham, MA) prior to downstream applications. The V4 region of the 16 s rRNA was amplified using adaptor and barcode-ligated specific primers [20, 21, 22]. Samples were sequenced using MiSeq Reagent Kit v2 chemistry, 500 cycles dual index 2 × 250 bp format (Illumina Inc., La Jolla, CA) according to manufacturer’s specifications.

### Quality Control and Processing of DNA Sequences

DNA sequences were processed to ensure that only quality sequences were applied to the mothur pipeline [23]. Stringent settings were maintained to ensure that there were no barcode mismatches among the demultiplexed reads. Sequence filtering was used by applying the screen.seqs function of mothur to remove all sequences shorter than 220 bp. Additional QC steps were implemented, and the sequences were aligned against the SILVA database to confirm the orientation of noise-filtered sequences and to ensure the correct positioning of the amplified and sequenced variable region reads [24]. The sequences passing QC were then checked for chimeras and classified taxonomically using mothur, eliminating hits matching mitochondria, chloroplast, archaea, eukaryote, and other unknown sequences, to avoid noise from the data [23]. Archaea was removed primarily due to poor taxonomic classification below the Phylum level using the SILVA database. Sequence reads were then clustered at various taxonomic levels including 97% rRNA sequence similarity defined as operational taxonomic units (OTU).

### Data Analysis

Species, genus, and phyla count tables of 16S rRNA reads from the mothur output files were used for all subsequent statistical analyses in the *R* statistical environment [25]. The VEGAN and APE *R* packages were used for all statistics calculations, multivariate analyses, including Bray-Curtis dissimilarity, PCoA, PERMANOVA, ANOSIM, and sample clustering [26, 27]. The mothur open-source software was also used to calculate Shannon and CHAO diversity indices to calculate species richness, and evenness. Kruskal-Wallis testing was used as a non-parametric approach to determine the statistical significance of the varying levels of abundance at the genus level between the samples. *P* values < 0.05 were considered significant in these calculations.

## Results

A total of 287,881 sequence reads from the artwork post filtering and quality control (QC) were generated; each sample swab averaged 7,500 sequence reads. As expected, several samples did not yield a significant number of sequence reads. These samples are suspected of having low microbial biomass upon collection. Samples with less than 2000 sequence reads, including the negative controls, were excluded in the analysis as this would have limited coverage and would not represent the microbial community as the other samples with higher coverage would. Sequences are publicly available in the National Center for Biotechnology Information (NCBI) Short Read Archive (SRA) under accession PRJNA505184.

### Microbial Composition Among Substrates: Wood, Canvas, and Marble

**Fig. 2:**
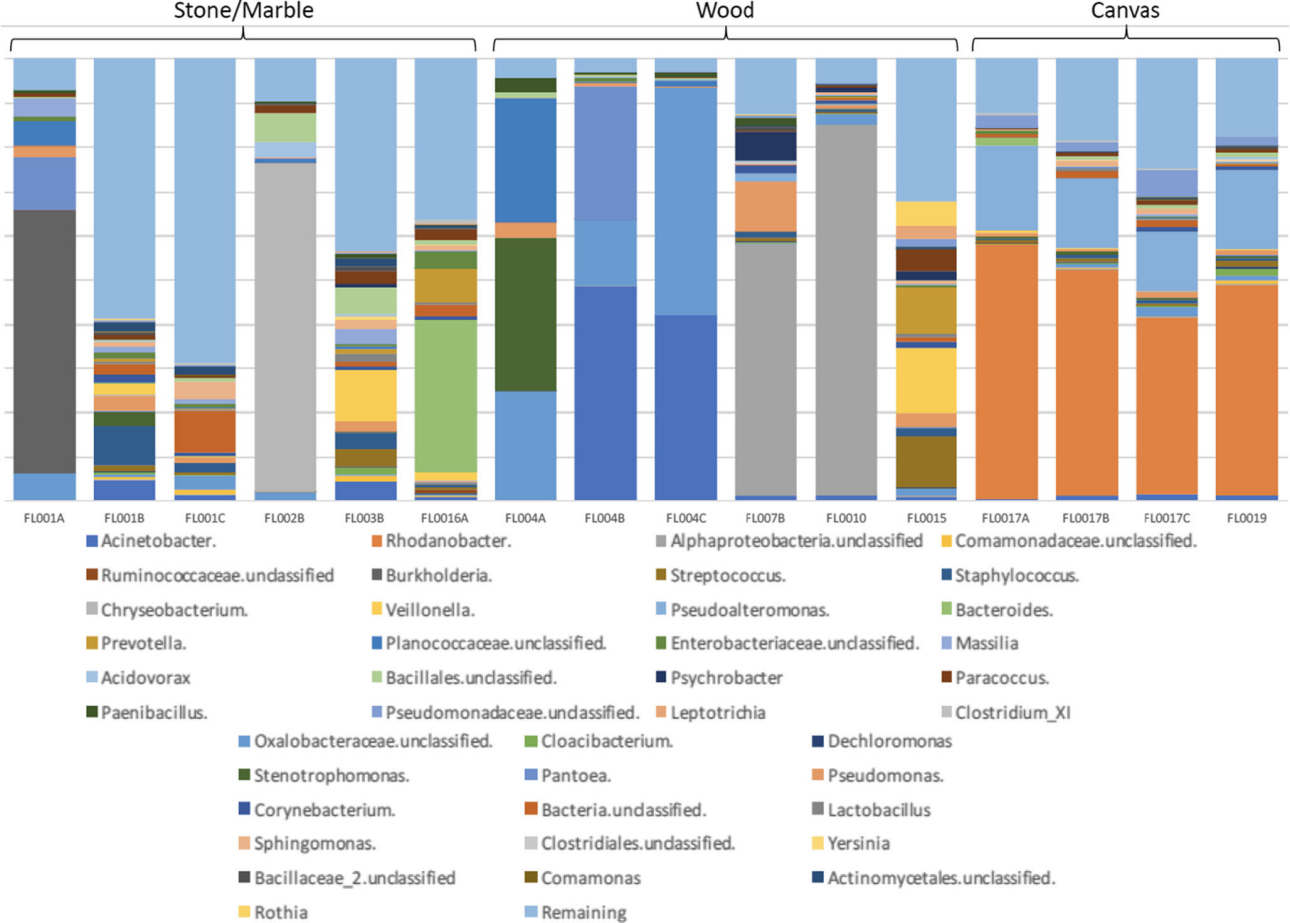
Stacked bar chart of the top 40 most abundant genera in the dataset. Sample types are grouped to show differences in taxonomy between substrate types as well as variation within sample types

**Fig. 3:**
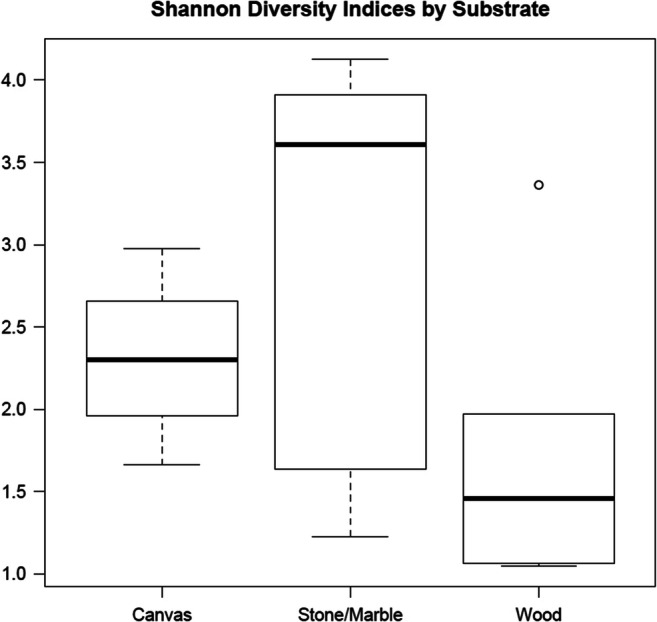
Shannon diversity index comparison between substrates to indicate the variation in microbial composition and richness between substrate types. Stone/marble appears to have the most diverse community found in our sample types

**Fig. 4:**
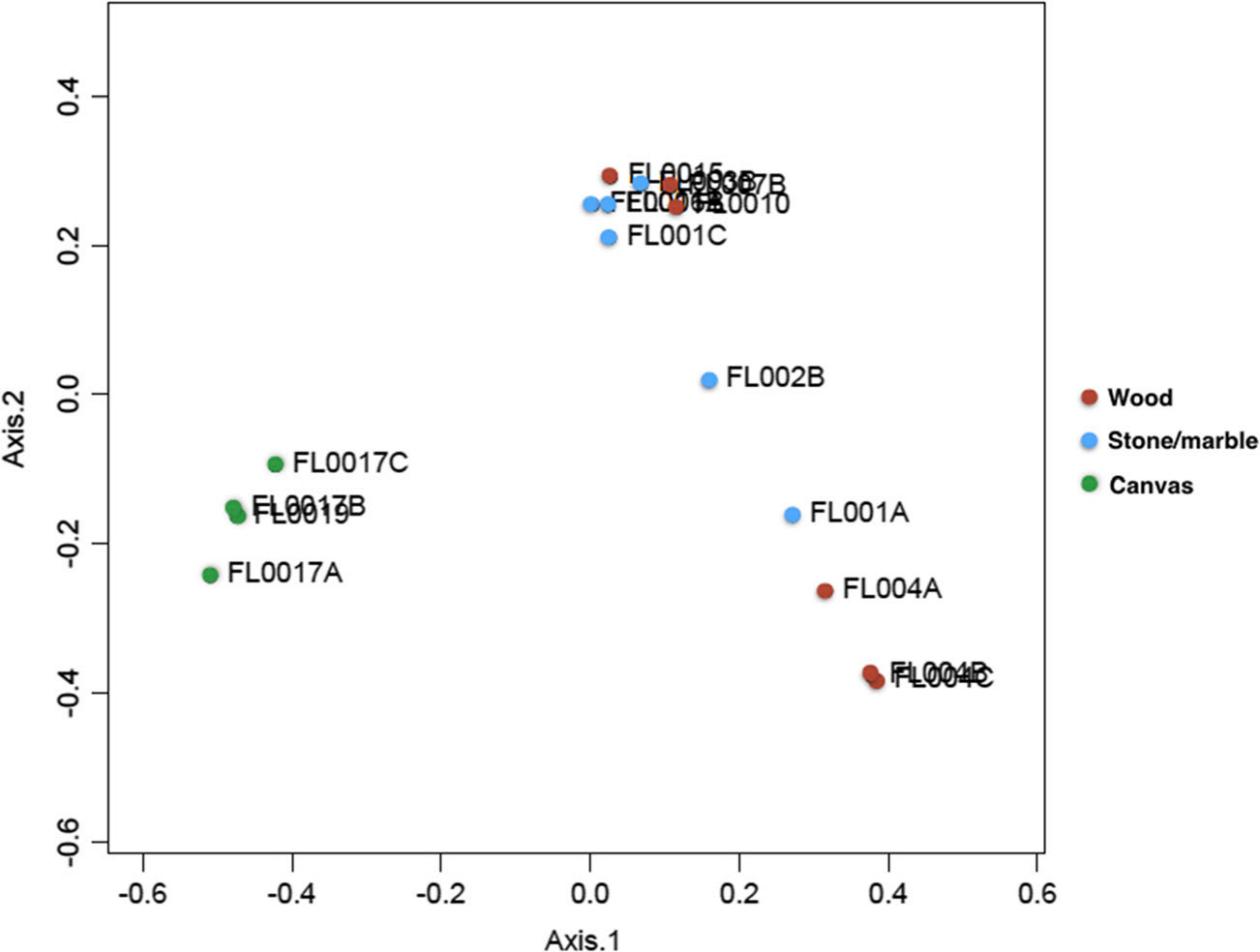
Principal coordinates analysis of sample swabs. Sample clustering is evident by sample type. Sample swabs collected from wood and stone were more similar to each other when compared to sample swabs collected from canvas

Upon review of the 16S data, the microbial populations were easily discernable between the different types of substrates sampled. Wood was primarily composed of unclassified *Oxalobacteraceae* and *Acinetobacter* (FL004A, B, and C) or *Alphaproteobacteria* (FL007B and FL0010). Paintings on canvas were primarily composed of *Rhodanobacter* and *Pseudoalteromonas*. Samples collected from paintings on canvas also showed the most consistency of which genera were most abundant regardless of the sample location or painting sampled (Fig. 2). The most abundant genera in stone/marble samples were unclassified *Oxalobacteraceae*, *Burkholderia*, *Staphylococcus*, *Pseudomonas*, *Bacteroidetes*, and *Chryseobacterium*. The microbial composition in stone/marble also varied more than wood and canvas, where swabs taken at different locations on the same artwork varied in microbial composition (Fig. 2). Furthermore, the stone/marble samples also had a higher degree of diversity when compared with canvas and wood samples (Fig. 3). Principal coordinates analyses indicate that the sample swabs can be distinguished according to the sample and substrate from which they were collected (Fig. 4).

### Microbial Population Diversity Decreases on Non-painted Wood when Compared with Painted Wood

**Fig. 5:**
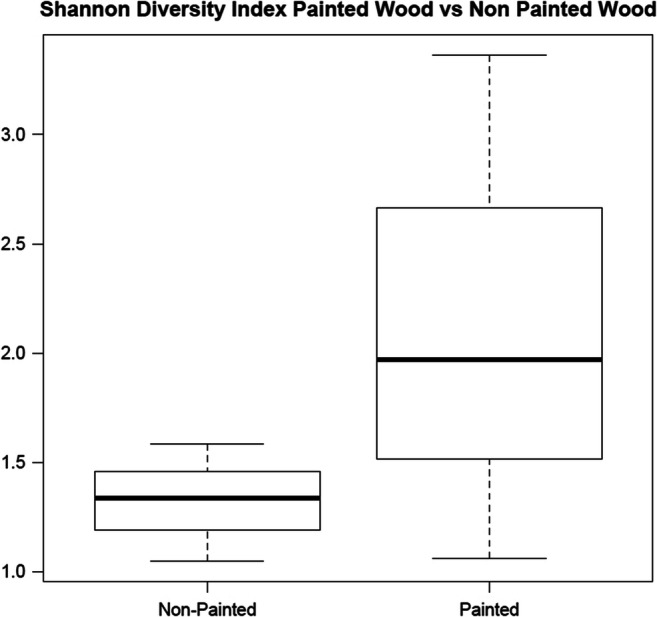
Shannon diversity index comparison between painted and non-painted wood. Larger overall spread of diversity indices summarizes the more complex communities in painted wood versus non-painted wood

Though there were some consistent results between sample swabs collected from within the same painting, there were differences in the microbial composition when comparing the ascension painting swabs (all from the same painting: FL007, FL010, and FL015) as compared with samples collected from a non-painted wood cabinet (FL004A-C) stored in the same vicinity. Non-painted wood sample swabs FL004A, FL004B, and FL004C were mostly abundant in *Acinetobacter* and unclassified *Oxalobacteraceae.* Painted wood sample swabs FL007 and FL010 were abundant in mostly unclassified *Alphaproteobacteria*, whereas FL015, also from the same painting, was composed of mostly *Streptococcus*, *Veillonella*, *Prevotella*, *Paracoccus*, and *Rothia*. Shannon diversity index calculations indicate that there is a higher level of diversity in microbial composition of painted wood when compared with non-painted wood (Fig. 5).

### Oxidase Positive Genera Present on Paintings on Canvas and Paintings on Wood

**Fig. 6:**
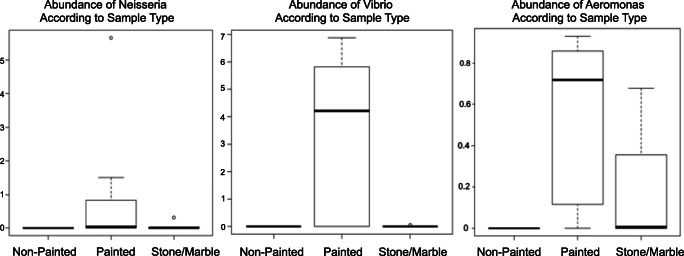
Comparing the varying abundances of specific genera in painted wood, non-painted wood, and stone/marble known to have various species capable of oxidase positive activity

We were able to detect the presence of five genera known to contain oxidase positive species in our dataset. *Pseudomonas* was detected in all samples regardless of substrate, and *Campylobacter* was detected at low frequencies in stone/marble and painted wood only. *Neisseria* and *Vibrio* were detected in higher abundance on painted wood when compared with stone/marble. Though *Aeromonas* was present on both painted wood and stone/marble, these genera was identified in higher abundance on painted wood when compared with stone/marble (Fig. 6).

## Discussion

The microbial signatures detected on the various pieces of artwork in our study provide us with a novel approach to not only characterizing the microbial composition on aging artwork but also insight into identification, restoration, and preservation efforts. We were able to easily distinguish the different sample types according to individual microbial biosignatures despite being stored within the same home in the private collection. The most interesting find in our study would be the presence of genera known to contain oxidase positive species found primarily on painted wood and canvas surfaces. This is of great interest to preservation efforts as members of oxidase positive species are capable of using oxygen for energy production and producing water or hydrogen peroxide as a byproduct. Such byproducts are likely to influence the presence of mold and the overall rate of deterioration. Additionally, oxidase positive bacteria have been known to be capable of metabolizing environmental contaminants rich in hydrocarbons, which are common in oil-based paints [28, 29]. *Pseudomonas* was identified in all sample types which was to be expected since this genus is a common environmental aerobe with diverse metabolic capabilities allowing various species to survive in different environments. The higher abundance of genera known to contain oxidase positive species; *Neisseria*, *Vibrio*, and *Aeromonas* in painted samples was of particular interest as they were specifically found on painted wood rather than non-painted wood. Currently, we are unable to speculate as to why this is the case until additional studies with higher numbers of samples and deeper sequencing are conducted for comparative analysis.

The varying surface types are likely to influence the microbial composition as all three substrates are different from each other when it comes to porosity, thickness, density, depth of crevices, and ability to retain moisture. When comparing the substrate types, it was interesting to see that the stone/marble samples showed greater microbial diversity when compared with wood and canvas. This is likely due to the porous nature of stone and marble which is harboring additional organisms and potentially moisture and nutrients, along with the likelihood of biofilm formation. The low microbial diversity observed in canvas and wood are likely the result of lack of nutrients as the primary source of energy would be the oil-based paint that few organisms can metabolize. Canvas and wood are also providing the microbial community additional nutrients as both substrates are high in cellulose and organic matter which are likely influencing the taxonomy of the populations found on these surfaces. Future studies should evaluate the metabolic process of highly abundant bacteria on wood and canvas surfaces.

Though our sample size is low, the novelty of our study has provided the art and scientific communities with evidence that microbial signatures are capable of differentiating artwork according to their substrate. In addition, we can also speculate that the origin of artwork can be recognized using similar techniques which would be exceedingly useful in confirming authenticity. In an unrelated study, we demonstrated that microbial signatures and patterns are geographically distinguishable when comparing the microbial signatures in human hairs collected near Washington D.C., and human hairs collected in San Diego, CA [30]. This approach can potentially be used to distinguish artwork from one location to another thus supporting any efforts of confirming authenticity and identifying any counterfeits that appear to originate in a different geographical location. Such approaches are well-utilized as many projects have focused on characterizing the microbial composition in regard to environmental and clinical studies, but has yet to be applied to this degree in regard to characterizing artwork [31, 32].

Enormous potential in preservation and restoration of artwork can also be achieved with these approaches as we have demonstrated that we are capable of identifying entire communities of bacteria present on various pieces of aging artwork. Future studies would benefit from working with samples whose authorship, ownership, and care are well-documented, although documentation about care of works of art (e.g., whether and how they were cleaned) seems rare before the mid-twentieth century. As we are currently proficient in identifying entire microbial communities on different surfaces, we provide a useful foundation for improving conservation and identification efforts. The next steps in this endeavor would be to characterize the metabolic processes that these communities use thus providing a clear understanding of microbial composition and function. Additional studies that incorporate genomics approaches to deteriorating artwork are necessary to fully characterize the microbial composition down to species and strain level taxonomy. Metagenomics and metatranscriptomics approaches will confirm the presence of metabolic genes responsible for oxidase activity, hydrocarbon metabolism, and upregulation of such processes respectively. Of particular interest would be presence and activity of oil degrading enzymes [28]. Such approaches will lead to fully understanding which organism(s) are responsible for the rapid decay of artwork while potentially using this information to target these organisms to prevent degradation. Focusing on reducing the abundance of such destructive organisms has great potential in preserving and restoring important pieces of human history.
